# Characterization of Peripheral Retinal Degenerations and Rhegmatogenous Lesions Using Ultra-Widefield Swept Source OCT Integrated with a Novel Scanning Laser Ophthalmoscope

**DOI:** 10.3390/diagnostics15222930

**Published:** 2025-11-20

**Authors:** Daniela Bacherini, Clara Rizzo, Giulio Vicini, Diego Luciani, Lorenzo Vannozzi, Gianni Virgili, Fabrizio Giansanti, Cristina Nicolosi

**Affiliations:** 1 Eye Clinic, Neuromuscular and Sense Organs Department, Careggi University Hospital, Largo Brambilla 3, 50134 Florence, Italy; daniela.bacherini@gmail.com (D.B.); giulio.vicini@gmail.com (G.V.); diegoluciani8@gmail.com (D.L.); oculistica.vannozzi@gmail.com (L.V.); gianni.virgili@unifi.it (G.V.); fabrizio.giansanti@unifi.it (F.G.); cristina.nicolosi@unifi.it (C.N.); 2 Department of Neurosciences, Psychology, Drug Research and Child Health, University of Florence, 50139 Florence, Italy

**Keywords:** peripheral retinal degenerations, rhegmatogenous lesions, ultra-widefield swept-source optical coherence tomography, OCT

## Abstract

**Background/Objectives**: The purpose of this study was to evaluate the implementation of ultra-widefield swept-source optical coherence tomography (SS-OCT) in characterizing peripheral retinal degenerations and rhegmatogenous lesions, and to assess its potential implications for clinical management. These lesions are often challenging to visualize with conventional techniques, highlighting the need for advanced imaging modalities to improve detection and characterization. **Methods**: We conducted a retrospective observational study involving patients diagnosed with peripheral retinal degenerations and/or rhegmatogenous lesions referred to our center. All participants underwent comprehensive ophthalmological evaluation, including slit-lamp biomicroscopy, dilated fundus examination, and peripheral SS-OCT imaging. Key parameters assessed included the presence of vitreoretinal attachment, vitreous traction, full-thickness retinal defects, and subretinal fluid associated with the peripheral lesions under investigation. **Results**: A total of 107 eyes from 95 patients were included. The mean spherical equivalent was −2.18 ± 2.5 diopters, and mean BCVA was 0.03 ± 0.11. Peripheral SS-OCT imaging successfully captured and characterized 130 retinal lesions, including retinal tears (*n* = 34), lattice degeneration (*n* = 25), retinal holes (*n* = 21), peripheral retinoschisis (*n* = 17), and schisis/detachment (*n* = 7). Less commonly observed lesions were snail track degeneration (*n* = 4), white without pressure (*n* = 4) microcystic degeneration (*n* = 2), dialysis (*n* = 2), condensed vitreous (*n* = 2), and paving stone degeneration (*n* = 1). SS-OCT provided high-resolution visualization of the peripheral retina and vitreoretinal interface, revealing findings such as vitreous traction, everted edges in retinal holes, and associated subretinal fluid, some of which were not clinically detectable and, in several cases, directly influenced management decisions. **Conclusions**: Ultra-widefield SS-OCT significantly enhanced the visualization of peripheral retinal degenerations and rhegmatogenous lesions, providing clinically meaningful details that may influence diagnosis and clinical decision-making.

## 1. Introduction

Since its introduction in 1991, optical coherence tomography (OCT) has proved to be a fundamental tool for the diagnosis and management of various macular diseases. It is now considered an essential instrument in ophthalmological clinical practice for the diagnosis and monitoring of several macular disorders, such as age related macular degeneration (AMD), vitreo-macular interface pathologies, macular edema and many others [1,2]. Although conventional OCT devices are limited to imaging only the central retina, recent advancements have led to the introduction of wide-field (WF) and ultra-wide-field (UWF) imaging systems, which allow visualization of up to 50° and 200° of the retina [3], respectively. Imaging of the retinal periphery has since gained increasing importance in the characterization and management of various vitreo-retinal diseases as retinoschisis, retinal detachment and peripheral degenerations, and other peripheral pathologies [4,5,6,7,8,9]. The ongoing development of UWF-OCT, with its ability to capture retinal structures extending to the far periphery, including the anterior edge of the vortex vein ampulla and beyond to pars plana, has drawn the interest of vitreoretinal experts enabling a more accurate understanding of the pathology and of the structural abnormalities behind peripheral retinal lesions. Indeed, the characterization of specific peripheral retinal areas with UWF OCT imaging may provide insights that directly affect clinical management decisions, such as choosing whether to monitor or treat peripheral retinal findings. However, despite its potential, there is still limited literature assessing peripheral retinal degenerations and rhegmatogenous lesions through UWF-OCT. In this study, we aimed to characterize peripheral degenerations and rhegmatogenous retinal lesions, using the Optos Silverstone (Optos PLC; Dunfermline, UK), a multimodal imaging platform that allows the acquisition of UWF swept-source OCT (SS-OCT) scans of the extreme retinal periphery. We evaluated key features, including the analysis of the vitreoretinal interface and the presence of vitreoretinal traction, as well as associated characteristics such as full-thickness defects, subretinal fluid, localized retinal thinning. The study also aimed to assess the feasibility of using this device in routine clinical practice for the visualization of peripheral lesions and to explore its potential impact on clinical decision-making.

## 2. Material and Methods

We performed a retrospective observational study on 95 patients with previously diagnosed peripheral retinal degenerations and/or rhegmatogenous lesions, referred to the Eye Clinic, Careggi University Hospital, in Florence (Italy) between October 2022 and October 2024. The study conformed to declarations of Helsinki. Informed consent was obtained from all the patients. Patients aged over 18 years who were found to have peripheral retinal degenerations or rhegmatogenous lesions on indirect ophthalmoscopy were included in the study. All patients had been evaluated in our medical–surgical retina outpatient clinic, where they were referred for various retinal or macular concerns, including peripheral degenerations, suspected rhegmatogenous lesions or other vitreoretinal abnormalities. Patients with media opacities or uncooperative behavior that impeded proper image acquisition were excluded. Demographic data (age, sex) and clinical characteristics (refractive error, lens status, and symptoms) were collected from patient charts. Each patient underwent a complete ophthalmological examination with measurement of best corrected visual acuity (BCVA), slit-lamp biomicroscopy, dilated fundus examination, and multimodal imaging assessment, including peripheral SS-OCT (Optos Silverstone) (Optos PLC; Dunfermline, UK). The Optos Silverstone SS-OCT is a new multimodal imaging platform that allows the acquisition of SS-OCT scans of the extreme periphery. It produces an initial high-resolution scanning laser ophthalmoscope (SLO) fundus image up to 200° followed by navigated SS-OCT line or volume scans of the far periphery guided by the ultra-widefield SLO image. The system employs a 1050 nm light source with an axial resolution <7 μm and transverse resolution <20 μm, allowing deep tissue penetration and high-definition imaging of the retina from the vitreous to the choroidal–scleral interface in less than 0.5 s. Optomap-guided scans included line scans (6, 14, and 23 mm widths) and volume scans (heights ranging from 3 to 9 mm and widths from 6 to 14 mm). In this manuscript, the term UWF SS-OCT will refer to SS-OCT scans navigated to the peripheral retina using the ultra-widefield SLO image, while the OCT scan area itself remains limited to standard dimensions. SS-OCT imaging was performed by two experienced retinal imaging specialists (D.B., C.N.). At baseline, color optomap images and ultra-widefield 6 mm line and 6 mm volume OCT scans were obtained for all patients. Additional 6 mm HD volume scans, 23 mm extended line scans, and autofluorescence images were acquired at the discretion of the photographer. Images were assessed for diagnostic confirmation, lesion localization, and image quality. Peripheral retinal degenerations were classified according to the International Wide field Imaging Study Group [6] guidelines: the mid-periphery was defined as the retina from the vascular arcades to the posterior edge of the vortex vein ampullae, and the far periphery as the retina anterior to the vortex vein ampullae. UWF optomap and SS-OCT findings were analyzed focusing on the presence or absence of full-thickness defects, subretinal fluid, vitreoretinal traction, edge morphology in retinal holes, other associated features, and the presence or absence of posterior vitreous detachment (PVD).

## 3. Results

A total of 107 eyes from 95 patients with peripheral retinal degenerations and/or rhegmatogenous lesions were scanned using the Optos Silverstone platform. The mean age of the study population was 50.9 ± 15 years. A total of 46 men and 49 women were included. The mean refractive error (spherical equivalent) was −2.18 ± 2.5 diopters (range: −7.50 to 0). The median logMAR BCVA was 0.0, and the mean BCVA was 0.03 ± 0.11. Ninety-one patients were phakic, and four were pseudophakic. The baseline demographics of the study population are summarized in Table 1.

A total of 130 lesions were imaged. Among these, 12 different types of peripheral lesions were identified (Table 2). High quality images of the peripheral retina and vitreoretinal interface were obtained in all cases. The most prevalent peripheral lesions observed were retinal tears (34 out of 130) and lattice degenerations (25 out of 130 lesions). Other commonly observed lesions included retinal holes (21), peripheral retinoschisis (17) and retinal tufts (11). Less frequent lesions were schisis/detachment (7), snail track degeneration (4), white without pressure (4) microcystic degeneration (2), dialysis (2), condensed vitreous (2), paving stone degeneration (1). A total of 45 degenerations were classified as mid-peripheral and 85 as peripheral. Eighteen lesions occurred in highly myopic eyes (myopia > 6 diopters). Six lesions were identified in eyes with a history of ocular trauma.

The UWF SS-OCT characteristics of the most common peripheral retinal lesions are detailed in the paragraphs below.

Lattice degeneration (*N* = 25)

**Figure 1:**
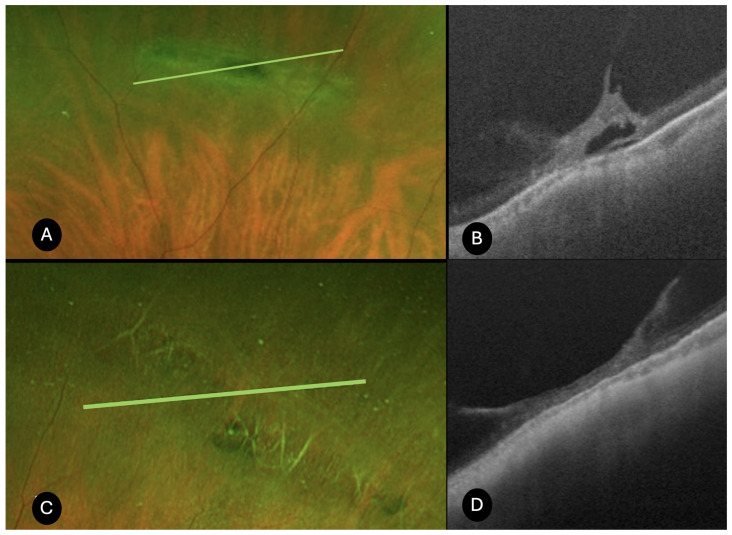
UWF SS-OCT findings in lattice degeneration. (**A**,**C**) Retinography of a lattice degeneration. (**B**) SS-OCT shows vitreous traction, hyperreflective deposits, and localized subretinal fluid associated with the lattice lesions. (**D**) Diffuse vitreous traction over lattice degeneration, and choroid thinning are visible.

On UWF color optomap, the lesions appeared as elongated, whitish peripheral alterations, typically aligned parallel to the ora serrata, with attenuated underlying blood vessels traversing the lattice lesions. A total of 12 lesions exhibited a focal shape, while 13 displayed a linear configuration. UWF SS-OCT revealed irregular thinning of the neuroepithelium in all cases (Figure 1). Full-thickness retinal holes were identified in 21 cases, whereas retinal tears were present in 2 cases, with subretinal fluid detected in 4 cases. Additionally, OCT demonstrated microcystic changes in the perilesional retina in 4 cases. Peripheral OCT also provided valuable insights into the vitreoretinal interface, showing vitreous liquefaction over the lattice lesions in 19 cases. Vitreoretinal traction was observed in 22 cases (focal in 9 cases and diffuse in 13). Hyperreflective deposits within the retina were noted in 3 cases, while irregularities of the retinal pigment epithelium were identified in 20 cases. Choroidal thinning was detected in 19 cases, and posterior vitreous detachment was observed in 8 cases. Prophylactic laser photocoagulation was performed in the 2 cases with associated retinal tears, and in 8 additional cases presenting with full-thickness retinal holes accompanied by vitreoretinal traction and photopsias.

Retinal Cystic Tufts (*N* = 11)

**Figure 2:**
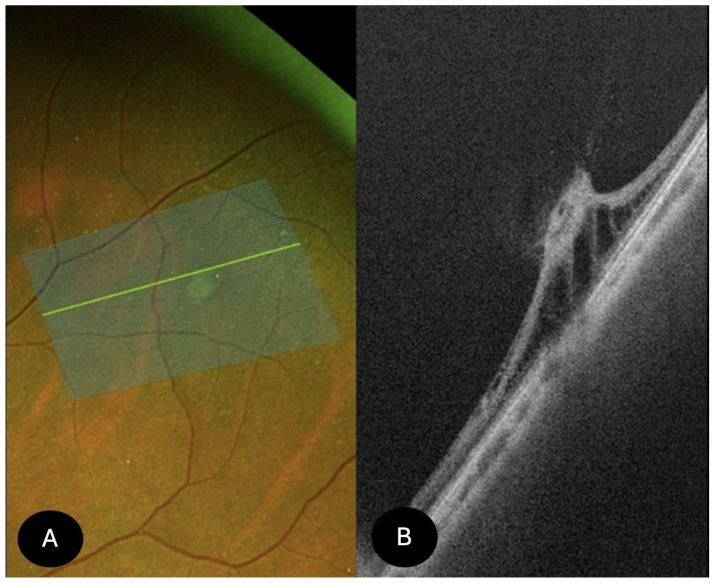
UWF SS-OCT findings in cystic tuft. (**A**) Retinography of the lesion which appears small and circumscribed, with a chalky-white color. (**B**) An hyperreflective irregular elevated lesion with internal hypo reflective cystoid or schisis-like cavities is depicted in UWF SS-OCT. Normal layers of the neuroepithelium are not distinguishable. Vitreous traction is evident.

Cystic retinal tufts appeared on UWF color optomap as round or oval, slightly elevated vitreoretinal degenerations. These lesions were small, well-circumscribed, and displayed a chalky-white coloration, with vitreous attachment to their surface. UWF SS-OCT scans revealed hyperreflective, irregularly elevated lesions containing internal hyporeflective cystoid cavities, with indistinguishable neuroepithelial layers. In all cases, peripheral SS-OCT also demonstrated vitreous traction at the apex of the lesion and vitreous condensation (Figure 2). Choroidal thinning was observed in eight cases, while vitreous lacunae were clearly visible in five cases. Full-thickness retinal defects were associated in three cases, and posterior vitreous detachment was detected in two cases.

Peripheral Retinal Holes (*N* = 21)

**Figure 3:**
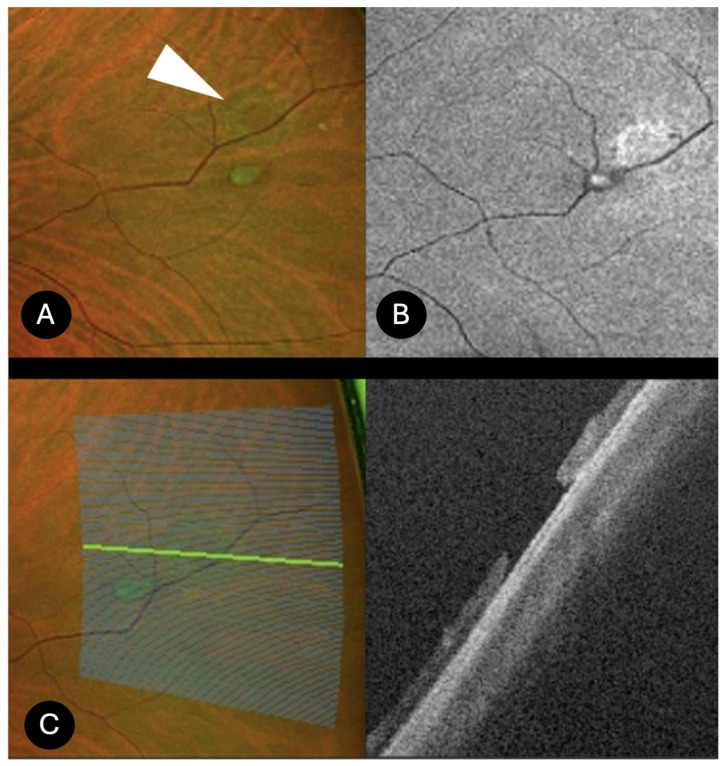
UWF SS-OCT of peripheral retinal hole. Retinography of a retinal peripheral hole (white arrow) with an associated operculum (**A**), autofluorescence (**B**). Peripheral OCT showing slightly elevated borders of the hole with no vitreous traction (**C**).

**Figure 4:**
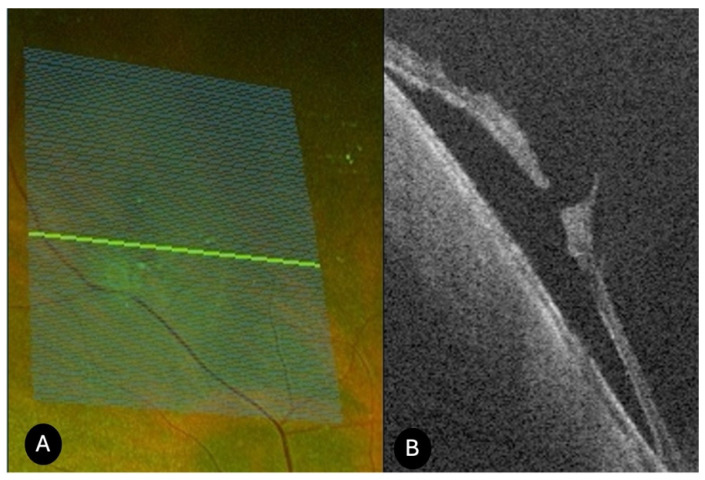
UWF SS-OCT of peripheral retinal holes. (**A**) retinography of a peripheral retinal hole; (**B**) peripheral retinal hole with associated subretinal fluid. Elevated borders of the hole and vitreous traction at the border of the hole are appreciable. The patient reported phosphenes, consistent with the presence of vitreoretinal traction observed on OCT.

Peripheral retinal holes appeared on UWF retinography as round or oval full-thickness retinal defects. UWF SS-OCT revealed everted edges in 15 cases, while in the remaining cases the edges were flat; in 10 cases, an operculum was observed in the adjacent vitreous (Figure 3). Retinal pigment epithelium (RPE) irregular thickening with uneven deposits was noted in 9 cases, and in 6 cases a circumscribed shallow retinal detachment was associated (Figure 4). Retinal microcystic changes were present in 9 cases. Vitreous traction at the inner retinal surface was appreciable in 13 cases, whereas no vitreous traction was detected in the others. A complete posterior vitreous detachment was present in 9 cases, while hyaloid detachment at the level of the macula was recorded in 12 cases. Seven patients reported phosphenes, and 8 patients complained of floaters (myodesopsia). All seven patients with photopsias exhibited vitreoretinal traction on OCT, while 6 out of 8 patients reporting floaters had an associated operculum. In 15 cases, prophylactic laser photocoagulation was performed based on SS-OCT evidence of vitreoretinal traction at the hole margins and associated subretinal fluid, even in the absence of symptoms, due to the structural features indicative of increased risk for progression to retinal detachment. Conversely, no laser treatment was performed in cases of retinal holes with flat edges and no associated traction and symptoms.

Snail track degeneration (*N* = 4)

**Figure 5:**
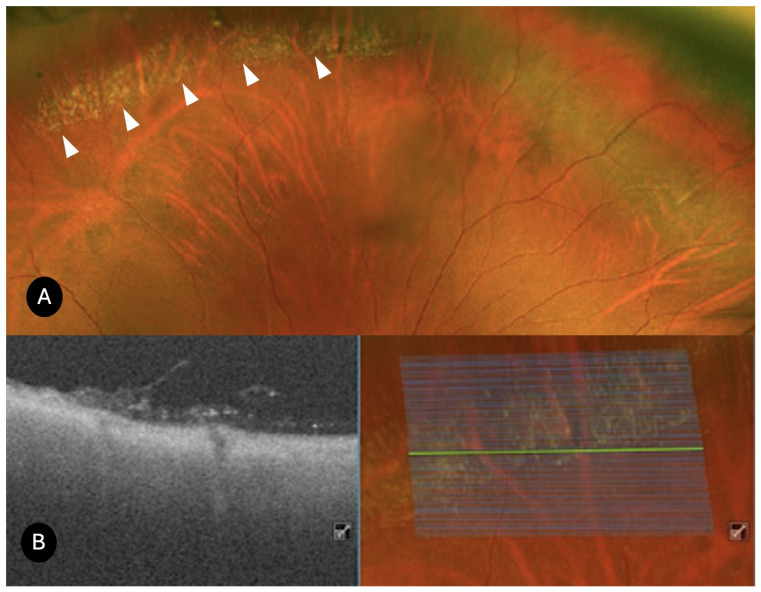
UWF SS-OCT findings in snail track degeneration. (**A**) retinography image showing the snail track degeneration (white arrows); (**B**) OCT findings showing vitreous traction, irregular retinal thinning, and hyperreflective retinal deposits.

In UWF color optomap, snail track degeneration appeared as linear bands of retina composed of tightly packed white dots resembling snowflakes. UWF SS-OCT revealed irregular retinal thinning and hyperreflective inner retinal dots in all lesions (Figure 5). Additional OCT findings included diffuse vitreous traction in four cases and cystoid changes with full-thickness retinal holes in two cases. All patients were asymptomatic and prophylactic laser photocoagulation was not performed in any case.

White Without Pressure (*N* = 4)

**Figure 6:**
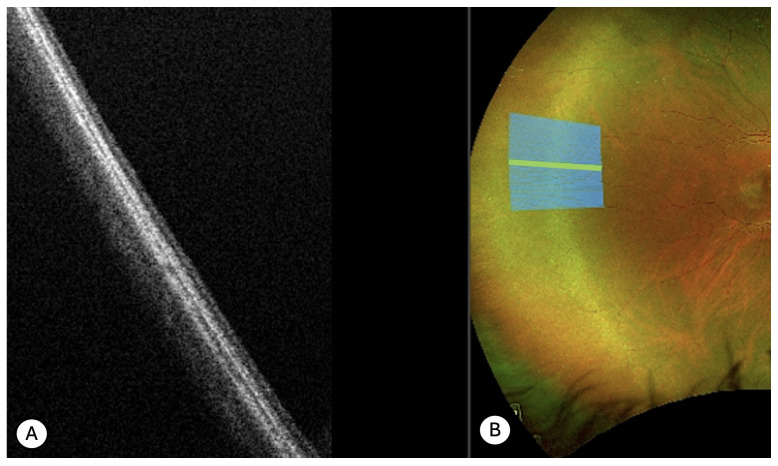
UWF SS-OCT in white without pressure degenerations. (**A**) peripheral SS-OCT characterized by hyperreflective outer retinal layers and ellipsoid zone, without vitreous traction. (**B**) Retinography of the white without pressure appearing as irregular, translucent white areas.

White without pressure degenerations appeared on UWF color optomap as irregular, translucent white areas. On peripheral SS-OCT, these lesions were characterized by hyperreflective outer retinal layers and ellipsoid zone, without evidence of vitreous traction (Figure 6).

Peripheral retinoschisis (*N* = 24)

**Figure 7:**
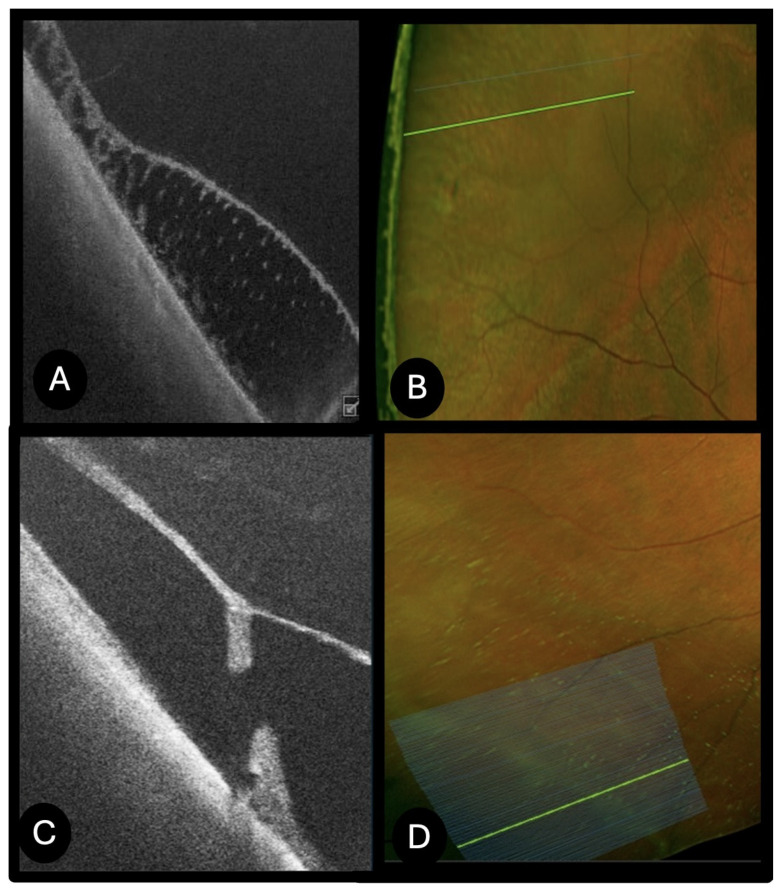
UWF SS-OCT findings in peripheral retinoschisis. (**A**) UWF SS-OCT showing a typical “saw-tooth” schisis-like separation of the retinal layers, with intraretinal columns. (**B**) Retinography of (**A**); (**C**) UWF SS-OCT showed a splitting into inner retinal layers and external retinal tear (**D**) retinography of (**C**).

On UWF retinography, peripheral retinoschisis appeared as a dome-shaped lesion with a translucent inner retinal surface. Peripheral SS-OCT revealed retinal splitting into multiple layers, predominantly involving the inner retina, with a characteristic “saw-tooth” schisis-like separation, intraretinal columns, and preservation of the retinal pigment epithelium (Figure 7). Among the 24 cases imaged with the Optos Silverstone, an associated detachment was present in 7 cases. Breaks at the level of the external schisis layer were detected in 5 cases. Intraretinal hyporeflective cavities dividing the neurosensory retina into inner and outer layers, along with photoreceptor alterations and disruption, were also observed. Overlying vitreous condensation was seen in 16 cases, and vitreoretinal traction was visible in 8 cases. Schisis-associated retinal tears with pigment epithelium elevation (schisis-detachment) were identified on SS-OCT in 5 cases. Complete posterior vitreous detachment was recorded in 6 cases, while posterior hyaloid detachment was documented in 15 cases. OCT image inversion artifacts occurred in 15 cases. Clinically, 2 patients reported photopsias, 2 reported floaters (myodesopsia), and most cases were asymptomatic. Six cases required vitreoretinal surgery.

Retinal tears (*N* = 34)

**Figure 8:**
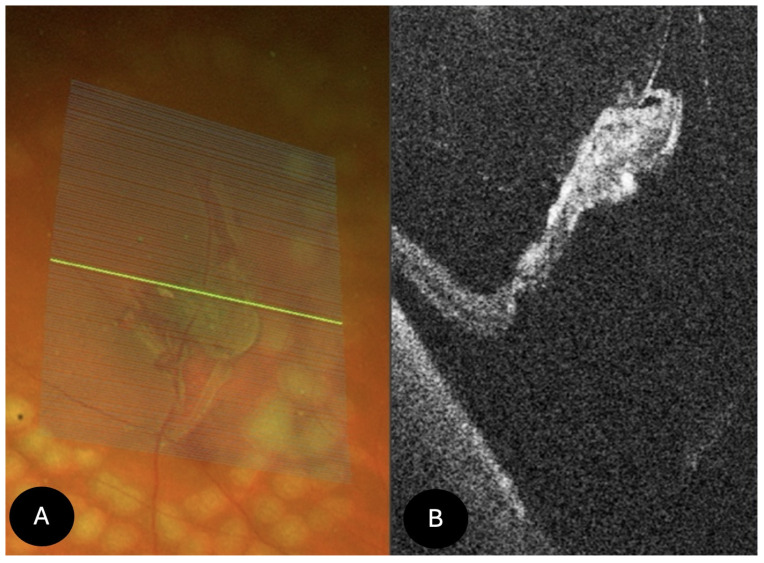
UWF SS-OCT findings in retinal tears. (**A**) perivascular retinal tear, already treated with laser. (**B**) OCT shows a shallow retinal detachment, hyperreflectivity on the edge of the tear, and vitreous traction.

We analyzed 34 retinal tears (horseshoe tears), which appeared on SS-OCT as full-thickness retinal defects with a partially detached flap, within which vitreous insertion was visible (Figure 8). Fifteen tears were associated with subretinal fluid or subclinical retinal detachment and exhibited either a partially attached or detached flap. In eyes with an attached flap, hyperreflectivity of the vitreous adherent to the inner retinal surface was observed. In 26 cases, imaging was performed after laser treatment. The retinal pigment epithelium demonstrated focal hyperreflective thickening in 16 cases. Posterior vitreous detachment was detected in 25 cases. Symptomatically, 14 patients reported photopsias, while 20 patients complained of floaters (myodesopsia). All cases underwent prophylactic laser treatment.

Retinal dialysis (*N* = 2)

We studied two cases of retinal dialysis, one of which was treated with laser photocoagulation. Retinal dialysis appeared as a circumferential retinal break located at the ora serrata. SS-OCT demonstrated vitreoretinal traction with retinal elevation, rolled edges, and complete absence of the retina distal to the break, which is consistent with the diagnosis of dialysis.

Microcystic degeneration (*N* = 2)

**Figure 9:**
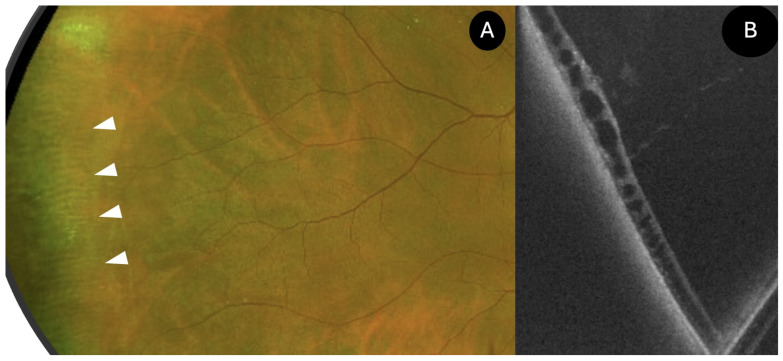
UWF SS-OCT findings in microcystic degeneration. (**A**) UWF retinography shows clusters of tiny translucent vesicles and fine radiating lines on a grayish-white peripheral background. (**B**) SS-OCT demonstrates characteristic sawtooth-like patterns composed of hyporeflective cystoid cavities and broad vertical columns extending through the entire neuoretina, consistent with microcystic degeneration.

We analyzed two cases of microcystic degeneration. On UWF retinography, they appeared as a cluster of tiny vesicles or vacuoles on a grayish-white background. SS-OCT revealed characteristic sawtooth patterns composed of hyporeflective cystoid cavities and broad vertical columns spanning the entire neuroretina (Figure 9).

## 4. Discussion

Peripheral retinal degenerations are common findings in clinical practice, frequently identified during dilated fundus examination of both symptomatic and asymptomatic patients. While many of these lesions do not carry a significant risk of rhegmatogenous retinal detachment (RRD), specific subtypes such as lattice degeneration, degenerative retinoschisis, retinal tufts, and retinal holes have been associated with a higher predisposition to RRD [10,11,12]. The prophylactic treatment of retinal degenerations, through laser or cryotherapy, remains controversial and is not clearly defined by clinical recommendations. As a result, ophthalmologists focus on educating high-risk patients about the signs and symptoms of posterior vitreous detachment and advise them to seek immediate ophthalmological evaluation if these symptoms arise [10,11]. In our cohort, most degenerative lesions were asymptomatic and detected incidentally during routine examination, whereas symptoms such as photopsias or floaters were primarily reported in eyes with retinal tears or retinal holes, consistent with their tractional or full-thickness nature. The advent of UWF high-resolution retinal imaging has significantly improved the documentation, detection, and characterization of peripheral retinal lesions. Firstly, UWF-OCT has revolutionized peripheral retinal imaging by enabling precise morphological descriptions that may form the basis for a classification of peripheral retinal lesions. In our series, we identified two distinct SS-OCT patterns of peripheral retinal holes: holes with everted edges, subretinal fluid, cystoid changes in the surrounding retina, and vitreous adhesion at the inner retinal surface, often associated with a floating operculum; and holes characterized by localized retinal thinning, flat edges, occasional cystoid changes, and absence of vitreous adhesion or subretinal fluid. We propose that the first type represents tractional (non-atrophic) holes, often associated with focal vitreoretinal traction and operculated morphology, while the second type represents atrophic holes, likely resulting from progressive retinal thinning and carrying a lower risk of retinal detachment. These findings align with Choudhry et al. [6] who described operculated holes with vitreous adhesion and an inverted V-shaped OCT profile, in contrast to flat-profile holes lacking vitreous traction or subretinal fluid. Similarly, Govetto et al. [4] distinguished operculated from non-operculated holes and suggested that non-operculated holes often show vitreoschisis and tangential traction, making them less likely to be truly atrophic. Distinguishing tractional retinal tears from atrophic holes is clinically important because tractional tears tend to arise acutely from dynamic vitreoretinal forces, progress more rapidly toward rhegmatogenous retinal detachment, and frequently require urgent intervention. In contrast, atrophic holes typically develop slowly, remain stable over long periods, and carry a much lower risk of retinal detachment [13].

Secondly, OCT findings provide detailed information that correlates well with histological descriptions of peripheral lesions. Histology of lattice degeneration demonstrates liquefied vitreous overlying the lesion, firm vitreous adhesion at its margins, discontinuity of the internal limiting membrane, and atrophic inner retinal layers, often accompanied by lipid inclusions and intraretinal fibrils in glial cells [14], findings that are reflected in OCT imaging. Similarly, OCT scans of cystic retinal tufts correspond to their histological features, showing dome-shaped elevations with internal microcysts and glial nodules enclosing vitreous crypts. Histology also suggests no involvement of the vitreoretinal interface in snail-track degeneration [14], a characteristic consistent with our finding. Thirdly, peripheral OCT provides valuable insights regarding the vitreous and its relationship to the retina, providing more information than ophthalmoscopy in selected cases. In our study overlying vitreous liquefaction and firm adhesions at lesion margins, particularly in lattice degeneration, were typical findings that corroborate previous reports [15,16,17]. Similarly, our study of cystic retinal tufts through SS-OCT, revealed the presence of vitreous traction at the apex in all cases. Such observations explain the increased risk of tractional tears following posterior vitreous detachment in eyes with lattice degeneration and cystic tufts [18,19,20]. Conversely, white without pressure showed hyperreflective outer retinal layers and ellipsoid zone without vitreoretinal traction, consistent with previous studies [21,22], demonstrating its association with photoreceptor reflectivity changes rather than vitreoretinal traction. As a result, peripheral OCT finding can support consideration of prophylactic treatment in selected cases. In our study vitreous traction in retinal tears could also be clearly visualized on SS-OCT even when it was not evident clinically. As a matter of fact, peripheral OCT imaging can highlight subtle rhegmatogenous features not evident during clinical examination, such as vitreoretinal traction, everted edges on retinal holes, subretinal fluid, or microstructural breaks. In our study, for example, we identified multiple atrophic holes or tears in lattice degeneration through the use of UWF-OCT improving lesion detection compared to ophthalmoscopy and aiding in identifying those at greater risk for retinal detachment. Similarly, OCT proved highly effective in differentiating retinoschisis from retinal detachment by precisely visualizing breaks and associated detachment, in agreement with prior reports [23,24]. The medico-legal implications of these findings are significant. UWF-OCT enables objective documentation of peripheral lesions, particularly in cases referred for prophylactic laser treatment without clear rhegmatogenous features. The ability to provide objective, reproducible, and archivable documentation strengthens clinical decision-making and supports observation over unnecessary intervention. According to the American Academy Preferred Practice Pattern (PPP) [25], current management of peripheral retinal degenerations is largely based on ophthalmoscopic findings and patient-reported symptoms, with symptomatology remaining a key element in the decision-making process. However, OCT evaluation of the peripheral vitreoretinal interface may uncover subtle signs of traction or subretinal fluid that are not detectable with traditional examinations. While patient symptoms and indirect ophthalmoscopy with scleral indentation remain indispensable in the evaluation of peripheral retinal lesions, peripheral OCT adds substantial value by providing objective, reproducible, and documentable data. In our cohort in a small number of cases, we elected to perform prophylactic laser treatment even in asymptomatic eyes when UWF SS-OCT revealed clear vitreoretinal traction or subtle rhegmatogenous features, as these findings suggested a higher risk than was evident on clinical examination alone. Nevertheless, we must also consider the added value of peripheral OCT in providing objective, reproducible, and documentable data. This not only enhances clinical understanding but also allows for image archiving, which could prove extremely useful from a medico-legal standpoint particularly in asymptomatic patients or in ambiguous cases where treatment decisions must be supported with tangible evidence. Consequently, the presence or absence of vitreoretinal traction visible using OCT could redefine the classification of lesions such as holes and lattice degeneration, influencing both our diagnostic framework and therapeutic choices. We hypothesize that OCT-based assessment of the peripheral vitreous could become a valuable addition to existing paradigms, potentially leading to new therapeutic criteria, improved risk stratification, and ultimately more tailored patient care. This study has several limitations: its retrospective design, the limited number of eyes analyzed, and the cross-sectional nature of the study without a proper follow-up. An evaluation over time of specific structural characteristics, such as increased vitreous traction or changes in the amount of associated peripheral subretinal fluid, may assist ophthalmologists in determining when and if intervention is necessary. Further prospective comprehensive studies, which proper follow up are required to evaluate which specific OCT findings may correlate with a higher risk of rhegmatogenous retinal detachment.

## 5. Conclusions

In the present study, we evaluated peripheral retinal degenerations and rhegmatogenous lesions using UWF SS-OCT. This imaging approach provided high-quality, clinically meaningful visualization of the peripheral retina and vitreoretinal interface, allowing for detailed assessment of ultrastructural features such as subretinal fluid, full-thickness retinal defects, and vitreous traction. These findings can significantly influence clinical decision-making and reduce unnecessary interventions, ultimately contributing to more tailored and evidence-based management of patients at risk for RRD. Nevertheless, further prospective studies are warranted to determine which OCT-derived characteristics are most predictive of rhegmatogenous risk and to establish the potential role of peripheral SS-OCT in guiding clinical decision-making and optimizing patient outcomes.

## Figures and Tables

**Table 1:** Baseline demographics and clinical characteristics of patients included in the study.

Patients, Eyes	95 Patients (107 Eyes)
Age (years), mean ± SD	50.8 ± 14.8
Gender (M/F)	46 (48.4%)/49 (51.6%)
Refractive error (diopters), mean ± SD	−2.18 ± 2.5
Median BCVA (LogMAR)	0.0
Lens Status (phakia/pseudophakia)	91 (95.8%)/4 (4.2%)

BCVA = best corrected visual acuity; F = female; M = male; SD = standard deviation.

**Table 2:** Different types of peripheral retinal degenerations identified in our study. All lesions were first detected on ultra-widefield SLO imaging, while peripheral SS-OCT was used to characterize the structural features of each lesion when OCT acquisition was feasible.

Type of Retinal Degeneration	*n* (%)
Retinal tear	34 (26.2)
Lattice degeneration	25 (19.2)
Retinal hole	21 (16.2)
Peripheral Retinoschisis	17 (13.1)
Retinal tuft	11 (8.5)
Schisis/Detachment	7 (5.4)
Snail track degeneration	4 (3.1)
White without pressure	4 (3.1)
Dialysis	2 (1.5)
Condensed vitreous	2 (1.5)
Microcystic degeneration	2 (1.5)
Paving stone degeneration	1 (0.8)

## Data Availability

The data presented in this study are available on request from the corresponding author. The data (original imaging) are not publicly available due to privacy issues. Some of the data included in the submitted manuscript were also incorporated into a specialization thesis that was deposited in a university’s institutional repository. This material was not published in any peer-reviewed journal.
